# Bayesian linear mixed model with multiple random effects for family-based genetic studies

**DOI:** 10.3389/fgene.2023.1267704

**Published:** 2023-10-19

**Authors:** Yang Hai, Wenxuan Zhao, Qingyu Meng, Long Liu, Yalu Wen

**Affiliations:** ^1^ Department of Statistics, University of Auckland, Auckland, New Zealand; ^2^ Department of Health Statistics, School of Public Health, Shanxi Medical University, Taiyuan, China

**Keywords:** bayesian linear mixed model, family-based genetic study, rare variants, unknown genetic factors, common environmental risk factors

## Abstract

**Motivation:** Family-based study design is one of the popular designs used in genetic research, and the whole-genome sequencing data obtained from family-based studies offer many unique features for risk prediction studies. They can not only provide a more comprehensive view of many complex diseases, but also utilize information in the design to further improve the prediction accuracy. While promising, existing analytical methods often ignore the information embedded in the study design and overlook the predictive effects of rare variants, leading to a prediction model with sub-optimal performance.

**Results:** We proposed a Bayesian linear mixed model for the prediction analysis of sequencing data obtained from family-based studies. Our method can not only capture predictive effects from both common and rare variants, but also easily accommodate various disease model assumptions. It uses information embedded in the study design to form surrogates, where the predictive effects from unmeasured/unknown genetic and environmental risk factors can be modelled. Through extensive simulation studies and the analysis of sequencing data obtained from the Michigan State University Twin Registry study, we have demonstrated that the proposed method outperforms commonly adopted techniques.

**Availability:** R package is available at https://github.com/yhai943/FBLMM.

## Introduction

Family-based study (e.g., twin study) is one of the most popular designs used in genetic research, and it offers many unique features for risk prediction studies. For example, the relatedness among family members helps capture the predictive effects from unmeasured/unknown polygenic and shared environmental factors, and thus contributes additional information, beyond the measured data, for risk prediction studies (Ruderfer et al., 2010). Despite these advantages, few statistical methods are available for risk prediction research using family-based designs. The existing methods usually build risk prediction models based on genetic effects that are estimated with familial correlations adjusted for. For example (Meigs et al., 2008), developed a risk prediction model for family-based genetic studies, where the genotypic risk score is determined without considering the information in families (Ruderfer et al., 2010). presented a family-based liability threshold model and illustrated it in the analyses of Crohn’s disease. Although these methods have contributed to the advances of family-based risk prediction, they can lead to less accurate models when unmeasured genetic and/or shared environmental factors contribute significantly to disease risk. Moreover, the recent whole genome sequencing studies have demonstrated that rare variants can play a significant role in many common complex diseases, such as obesity, coronary heart disease, and drug addiction (Ramachandrappa et al., 2013; Peloso et al., 2014; Wang et al., 2014). Family-based design can enhance the chance of capturing the predictive effects from rare variants as they tend to be aggregated within family. However, existing prediction models do not utilize the design information and they simply extend models designed for population-based studies by adjusting correlations within the data. Therefore, it remains challenging for them to capture the predictive effects from rare variants, primarily due to their low minor allele frequencies (Mihaescu et al., 2013).

It has long been recognized that family history alone can greatly facilitate disease risk prediction. For many complex diseases (e.g., cardiovascular diseases and type II diabetes), individuals with a positive family history are usually classified as the population at high risk (Valdez et al., 2007; Marateb et al., 2018). Family history can be viewed as a surrogate that reflects the contributions of many known/unknown risk factors accumulated within a family. Evidences have shown that familial effects account for a significant amount of disease variability. For example (Chen et al., 2007), have shown that 33% of variance of spherical equivalent can be attributed to childhood environmental effects. Furthermore, genetic variants can account for a substantial proportion of heritability for human traits (Couillard et al., 2001; Dirani et al., 2006). For example, genetic factors can explain as much as 87% of the variation in the susceptibility to asthma in twins with positive family history (Laitinen et al., 1998; Lichtenstein et al., 2009) found that genetic heritability for bipolar disorder and schizophrenia was 59% and 64%, respectively. The familial aggregation for many complex diseases is mainly due to the relatedness in genetic and environmental factors among family members, which carry important information and can be used to further improve prediction accuracy. However, most existing analytical methods are developed by simply extending those models designed for population-based studies, where family correlations are first adjusted. For example (Meigs et al., 2008), built a risk prediction model for family-based genetic study, where the relatedness among family members is adjusted using a generalized estimating equation model. Although statistically valid and these methods could capture the predictive effects from those measured known risk factors, they are not capable of using family information as surrogates to account for unmeasured predictors (e.g., shared environmental risk factors).

Population-based whole-genome sequencing studies have shown that rare variants are associated with many complex human diseases (Dickson et al., 2010; Helgadottir et al., 2016), and they have great potential in explaining the missing heritability (Cruceanu et al., 2013). For example, recent study has reported that rare variants in renal salt handling genes have contributed to variation of blood pressure (Ji et al., 2008; Stefansson et al., 2008) found that rare variants are associated with schizophrenia and autism (Ionita-Laza and Ottman, 2011). showed that four rare variants in *IFIH1* gene can lower the risk of type 1 diabetes. Recent developments in prediction research have also shed light on the importance of rare variants in building an accurate prediction model. For example, the risk prediction model for coronary artery disease in European and South Asian populations was built with rare variants incorporated, and it yields improved predictive accuracy (Lali et al., 2020). Despite their importance, few methods designed for family-based studies have considered the contributions of rare variants in disease risk prediction. Recently, we developed a Bayesian linear mixed model with multiple random effects (denoted as BLMM) to predict disease risk for population-based studies, where both common and rare variants have been explicitly considered (Hai and Wen, 2020). We have showed that the BLMM can capture the predictive effects from rare variants and is robust against various disease models. Though promising, it was developed for population-based studies, and thus cannot make use of the information embedded in the family-based study design.

To address these limitations, we proposed a family-based Bayesian linear mixed model with multiple random effects (denoted as FBLMM) for the prediction analysis on sequencing data obtained from family-based genetic studies. The proposed FBLMM uses the correlations among family members to construct surrogates for unmeasured risk predictors, and it can account for the predictive effects from both common and rare variants. In the following sections, we first presented the details of the proposed model, and then conducted extensive simulation studies to evaluate its performance. Finally, we illustrated its application using the whole-genome exome data from Michigan State University Twin Registry study (Burt and Klump, 2012).

## Materials and methods

The proposed FBLMM is built using a similar idea in BLMM presented in (Hai and Wen, 2020), where we assume genetic similarities can lead to phenotypic similarities. Fundamentally different from existing methods that adjust for family correlations, we utilize the information embedded in the family-based study design to further improve the prediction accuracy. Given M genetic regions that can be defined using various criteria (e.g., gene and pathway), we form the FBLMM model as:

$$Y=X\beta +\sum_{m=1}^{M}g_{m}+f+\epsilon_{n}\;\text{with}\;\epsilon_{n}∼N\left(0,I\sigma_{\epsilon }^{2}\right),$$ (1)

where Y is the outcome; X is the genotypes for all common variants; and β is their corresponding effect. $g_{m}$ is the cumulative predictive effect from all measured predictors, including rare variants, on region m . f is the familial effect due to shared environmental factors and genetic relatedness, and I is an n×n identity matrix.

Similar to existing sparsity regression models (Carvalho et al., 2008; Zhou, Carbonetto, and Stephens, 2013), the Xβ is designed to capture the predictive effects from isolated markers. To tease out the impact of noise, we followed the same procedure in (Hai and Wen, 2020), instead of using the spike and slab prior that can lead to an underestimation of posterior variances for β (Carbonetto et al., 2012). We re-parameterized Xβ as XΓβ , where Γ= diag( γ ) and $\gamma ={\left(\gamma_{1},\gamma_{2},...,\gamma_{p}\right)}^{T}$ is a vector of binary variables indicating whether each genetic variant is predictive. We used the Bernoulli Gaussian distribution as the priors for β and γ (i.e., $\beta_{j}∼N\left(0,\sigma_{\beta }^{2}\right)$ and $\left.\gamma_{j}∼\text{Bernoulli}\left(\theta_{0}=0.1\right)\right)$ , and this allows to obtain an unbiased estimation of the posterior variance of β as well as achieving variable selection for β (Zhou, Carbonetto, and Stephens, 2013; Fernandes et al., 2017).

Similar to linear mixed models that assume the infinitesimal effects (VanRaden, 2008), the cumulative predictive effects from common and rare variants for region m are modeled via $g_{m}$ , where we set a multivariate normal prior for each region-based cumulative predictive effect as

$$\begin{array}{rr} g_{m}|K_{m} & ∼N\left(0,K_{m}\sigma_{m}^{2}\right)\;m=1,...,M\\ \sigma_{m}^{2} & ∼IG\left(a_{1},b_{1}\right). \end{array}$$ (2)

$K_{m}$ is the genetic similarity for region m and it is defined as $K_{m}=G_{m}W_{m}G_{m}^{T}/p_{m}$ , where $G_{m}$ is the genotype matrix for region m and $p_{m}$ is the number of genetic markers in the region. $W_{m}={\left(w_{1},w_{2},...,w_{p_{m}}\right)}^{T}$ is the pre-specified weights used to capture the contribution of rare variants. Similar to existing literature (Wu et al., 2011; Lee et al., 2012), we define the weighted sum statistics types (denoted as WSS) of weights as $w_{j}=\frac{1}{MAF_{j}\left(1-MAF_{j}\right)}$ , where $MAF_{j}$ is the minor allele frequency for the j th variant. The hyper-parameters of $a_{1}$ and $b_{1}$ are set to be 0.1 for all regions. To expedite its computation, we re-parameterized the cumulative predictive effects part with the slab and spike prior as

$$Y=X\Gamma \beta +\sum_{m=1}^{M}\left(Z_{m}r_{m}U_{m}\right)+f+\epsilon ,$$ (3)

where $U_{m}∼N\left(0,I\sigma_{m}^{2}\right)$ , $Z_{m}=Q_{m}\Lambda_{m}^{\frac{1}{2}}$ and $E\left(r_{m}=1\right)=℘\left(r_{m}\right)$ . The re-parameterization facilitates the selection of predictive regions (i.e., $r_{m}=1$ indicates the region is predictive), and the details of its derivations can be found in appendix A.

Mounting evidences suggest that there are familial aggregations for many complex traits (Laitinen et al., 1998; Couillard et al., 2001; Dirani et al., 2006; Lichtenstein et al., 2009), and the relatedness in genetic and environmental factors among family members are thought to be the main reasons for this aggregation. Therefore, we split the familiar effect f into predictive effects due to genetic correlation (denoted as $g_{f}$ ) and shared environmental factors (denoted as $e_{f}$ ). Model 3 can be written as

$$Y=X\Gamma \beta +\sum_{m=1}^{M}\left(Z_{m}r_{m}U_{m}\right)+g_{f}+e_{f}+\epsilon_{n}.$$ (4)

We set the prior for $g_{f}$ as

$$\begin{array}{rr} g_{f} & ∼N\left(0,K^{g_{f}}\sigma_{g_{f}}^{2}\right)\\ \sigma_{g_{f}}^{2} & ∼IG\left(a_{0f},b_{0f}\right) \end{array},$$ (5)

where $K^{g_{f}}$ is the theoretical kinship coefficient matrix. The $g_{f}$ uses the genetic correlation between family members to improve the prediction accuracy, and it can be viewed as a surrogate for those predictive but unmeasured genetic variants. To account for the impact of environmental factors, we assume all family members share the same environment (e.g., diet) and set $e_{f}$ as

$$\begin{array}{rr} e_{f} & ∼N\left(0,K^{e_{f}}\sigma_{e_{f}}^{2}\right) \end{array},$$ (6)

where $K^{e_{f}}$ is a block diagonal matrix with each block being a matrix with all elements equal to 1. The $e_{f}$ is designed to capture the predictive effects from shared environmental factors, and it can also be viewed as a surrogate for those unmeasured environmental predictors shared by family members. We used the idea from (Z. Chen and Dunson, 2003) and decomposed $K^{e_{f}}$ and $K^{g_{f}}$ as $K^{e_{f}}=Q_{ef}\Lambda_{ef}Q_{ef}^{T}$ and $K^{g_{f}}=Q_{gf}\Lambda_{gf}Q_{gf}^{T}$ , where $\Lambda_{ef}$ and $\Lambda_{gf}$ are diagonal matrices with eigenvalues on their diagonals, and $Q_{ef}$ and $Q_{gf}$ are matrices of the corresponding eigenvectors. Eq. 4 can be written as

$$Y=X\Gamma \beta +\sum_{m=1}^{M}\left(Z_{m}r_{m}U_{m}\right)+Z_{gf}U_{gf}+Z_{ef}U_{ef}+\epsilon_{n},$$ (7)

where $Z_{gf}=Q_{gf}\Lambda_{gf}^{\frac{1}{2}}$ and $Z_{ef}=Q_{ef}\Lambda_{ef}^{\frac{1}{2}}$ . We adopted the mean-field variational Bayes algorithm (VB) to estimate parameters for FBLMM. Let $\xi =\left(\beta ,\gamma ,U^{g},U_{gf},U_{ef},r,\sigma^{2},\sigma_{gf}^{2},\sigma_{ef}^{2},\sigma_{\epsilon }^{2}\right)$ denotes all parameters of interest, where $\gamma =\left(\gamma_{1},\cdots ,\gamma_{p}\right)$ , $U^{g}=\left(U_{1},\cdots ,U_{M}\right)$ , $r=\left(r_{1},\cdots ,r_{M}\right)$ , and $\sigma^{2}=\left(\sigma_{1}^{2},\ldots ,\sigma_{M}^{2}\right)$ . The goal is to obtain an optimal approximation $q\left(\xi \right)$ of the posterior distribution on ξ by maximizing the evidence lower bound (ELBO). In details, we iteratively update the approximated distributions for $q\left(\xi \right)$ as

$$\begin{array}{rr} q\left(\xi \right)= & q_{\beta }\times \prod_{j=1}^{p}q_{\gamma_{j}}\times \prod_{m=1}^{M}q_{U_{m}}\times \prod_{m=1}^{M}q_{r_{m}}\times q_{U_{gf}}\times q_{U_{ef}}\times \\ & \prod_{m=1}^{M}q_{\sigma_{m}^{2}}\times q_{\sigma_{gf}^{2}}\times q_{\sigma_{ef}^{2}}\times q_{\sigma_{\epsilon }^{2}}, \end{array}$$ (8)

where $q_{\beta }=N\left(M_{\beta },S_{\beta }\right)$ ; $q_{\gamma_{j}}=Bernoulli\left(\psi_{j}\right)$ ; $q_{U_{m}}=N\left(M_{m},S_{m}\right)$ ; $q_{r_{m}}=Bernoulli\left(\phi_{m}\right)$ ; $q_{U_{gf}}=N\left(M_{gf},S_{gf}\right)$ ; $q_{U_{ef}}=N\left(M_{ef},S_{ef}\right)$ ; $q_{\sigma_{m}^{2}}=IG\left(a_{m},b_{m}\right)$ ; $q_{\sigma_{gf}^{2}}=IG\left(a_{gf},b_{gf}\right)$ ; $q_{\sigma_{ef}^{2}}=IG\left(a_{ef},b_{ef}\right)$ ; and $q_{\sigma_{\epsilon }^{2}}=IG\left(a_{\epsilon },b_{\epsilon }\right)$ . Each parameter of ξ can be estimated by using the coordinate ascent algorithm, the estimating equations used to update the parameters are listed in appendix A.

**FIGURE 1:**
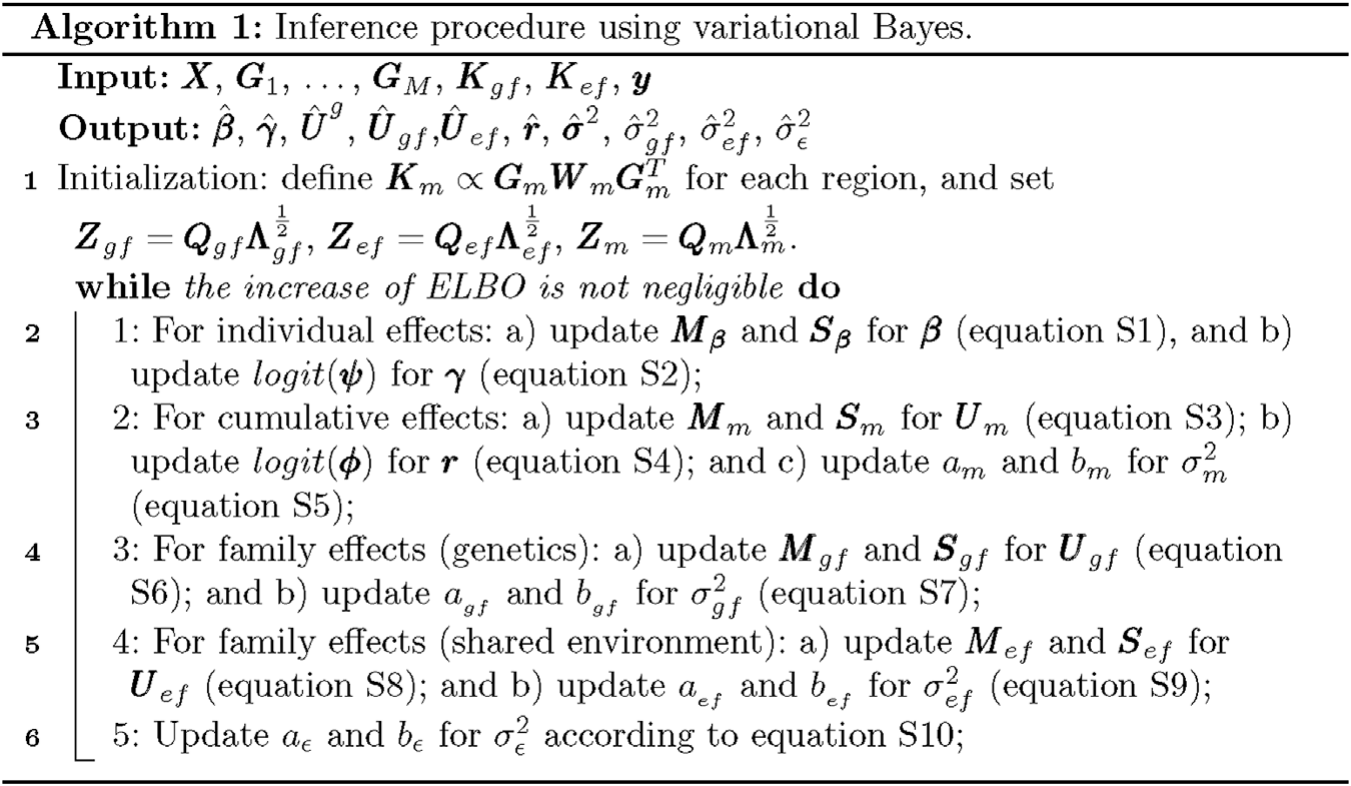
Algorithm 1: Inference procedure using variational Bayes.

The pseudo-code implementing our proposed model is shown in Figure 1. It is worth noting that when a new subject is not from families in the training data, its predicted value only depends on demographic and genetic predictors (i.e., the family information does not contribute to the outcomes). When a new individual comes from families in the training set, the FBLMM method not only uses genetic and demographic predictors, but also utilizes the extra information provided by family design to capture unmeasured genetic and shared environmental risk factors. Therefore, FBLMM has great potential to further improve predictions. The weight function employed by FBLMM can facilitate the identification of rare variants that are predictive, enabling FBLMM to consider contributions from both common and rare variants in prediction modeling.

## Simulation study

We conducted extensive simulation studies to evaluate the performance of our proposed method under various family-based designs, and further compared FBLMM with other widely used methods, including 1) adaptive MultiBLUP (Speed and Balding, 2014); 2) DPRVB (Zeng and Zhou, 2017); and 3) BLMM (Hai and Wen, 2020), where family correlations are first adjusted. Note that both MultiBLUP and DPRVB have shown to outperform other existing gBLUP-based methods (Speed and Balding, 2014; Zeng and Zhou, 2017).

To closely mimic the real human genome, the founders’ genotypes were drawn directly from Alzheimer’s Disease Neuroimaging Initiative (ADNI) study ( n=808 ). Pedigree simulator was used to simulate various types of pedigree structures and the gene-dropping method (Huang, Thomas, and Vieland, 2013) was implemented to generate the genotypes of offsprings. Each simulation scenario was replicated 100 times. We randomly split the simulated data into a testing set with 20% samples and a training set with the remaining 80% samples. Pearson correlations and root mean square errors (RMSE) that are calculated based on testing samples were reported for each method.

## Scenario 1: The impact of disease model

In this set of simulations, we evaluated the performance of our proposed method under three types of disease models, including outcomes are affected by 1) shared environmental factors only, 2) genetic factors only, and 3) both environmental and genetic factors.

### The outcome is affected by shared environmental factors only

To evaluate the impact of shared environmental factors, we randomly selected 3 genes from ADNI dataset and none of them was set to be causal. For simplicity and without loss of generality, we considered mixed two-generation pedigree structures, including a) half-sibling (Supplementary Figure S1A), parents with two offspring (Supplementary Figure S1B) and parents with four offspring (Supplementary Figure S1C). We used 808 samples from ADNI study as founders and formed a total of 394 families including 2040 individuals, which contained 150 individuals from 30 pedigrees of half-siblings, 708 individuals from 177 pedigrees of parents with two offsprings, and 1182 individuals from 197 pedigrees of parents with four offsprings. We simulated the outcomes as $Y_{ij}=\alpha_{i}+\epsilon_{ij}$ , where $\alpha_{i}$ is the shared environmental effects for family i and $\alpha_{i}∼N\left(0,\sigma_{a}^{2}\right)$ . It is straightforward to show that $Y∼N\left(0,K\sigma_{a}^{2}+I\sigma^{2}\right)$ , where K is a block diagonal matrix with each block being a matrix with all elements equal to 1. Therefore, we simulated the outcomes using $Y∼N\left(0,K\sigma_{a}^{2}+I\sigma^{2}\right)$ , where the percentage of the outcome variance explained by shared environmental factors increased from 25% to 75%.

**FIGURE 2:**
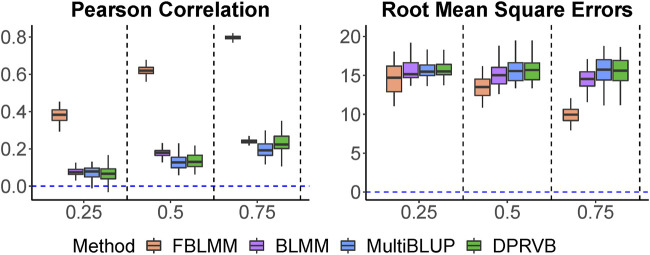
The comparison of prediction accuracy when outcomes are only impacted by shared environmental factors. The heritability increases from 25% to 75%.

Pearson correlations and RMSEs are shown in Figure 2. As expected, FBLMM significantly outperformed DPRVB, MultiBLUP and BLMM when shared environmental factors significantly contributed to disease risk. In addition, the prediction accuracy for FBLMM increases as the effects from shared environmental factors increase, but it remains almost unchanged for the other three methods. This is mainly because FBLMM is specifically designed to utilize information from family design for improved prediction. Although adjusting for the relatedness among family members makes it statistically valid to apply population-based methods on family-based studies, overlooking information embedded in the family design can lead to sub-optimal prediction performance. While DPRVB, MultiBLUP and BLMM have similar performance, BLMM tends to be slightly better. This is mainly because BLMM is flexible to the underlying disease models. While MultiBLUP assumes an infinitesimal effect model and DPRVB assumes an isolated effect model, BLMM-based method (i.e., BLMM and FBLMM) can easily accommodate these two commonly used model assumptions.

### The outcome is affected by genetic factors only

We evaluated the performance of FBLMM when only genetic variants, including both measured and unmeasured, contributed to the familial aggregation of traits. We first randomly selected three genes and set all of them as causal regions. We simulated the outcomes as $Y=\sum_{m=1}^{3}g_{m}+\epsilon$ , where $g_{m}$ is the genetic effect for region m and $g_{m}∼N\left(0,K_{m}\sigma_{m}^{2}\right)$ . $G_{m}$ is an $n\times p_{m}$ matrix of genetic markers on gene m and $K_{m}=\left(G_{m}W_{m}G_{m}^{T}\right)/p_{m}$ . Causal genetic variants can be unmeasured in practice (Wen, and Lu, 2017). Therefore, we randomly selected one of the three causal genes as unmeasured (i.e., only two causal genes are in the final simulated dataset). We set the total heritability to be 60% with the proportion of heritability accounted by unmeasured variants changing from 25% to 75%. To evaluate the performance of FBLMM across a range of phenotypes, we first considered the case where outcomes were mainly caused by common variants, and set $w_{j}=1$ for each predictor. We then simulated the cases where rare variants contributed substantially to disease risk. We simulated two models under such settings, where a beta-type of weights (denoted as BETA) $w_{j}=dbeta{\left(MAF_{j},1,25\right)}^{2}$ and a weighted sum statistics type of weights were used.

**FIGURE 3:**
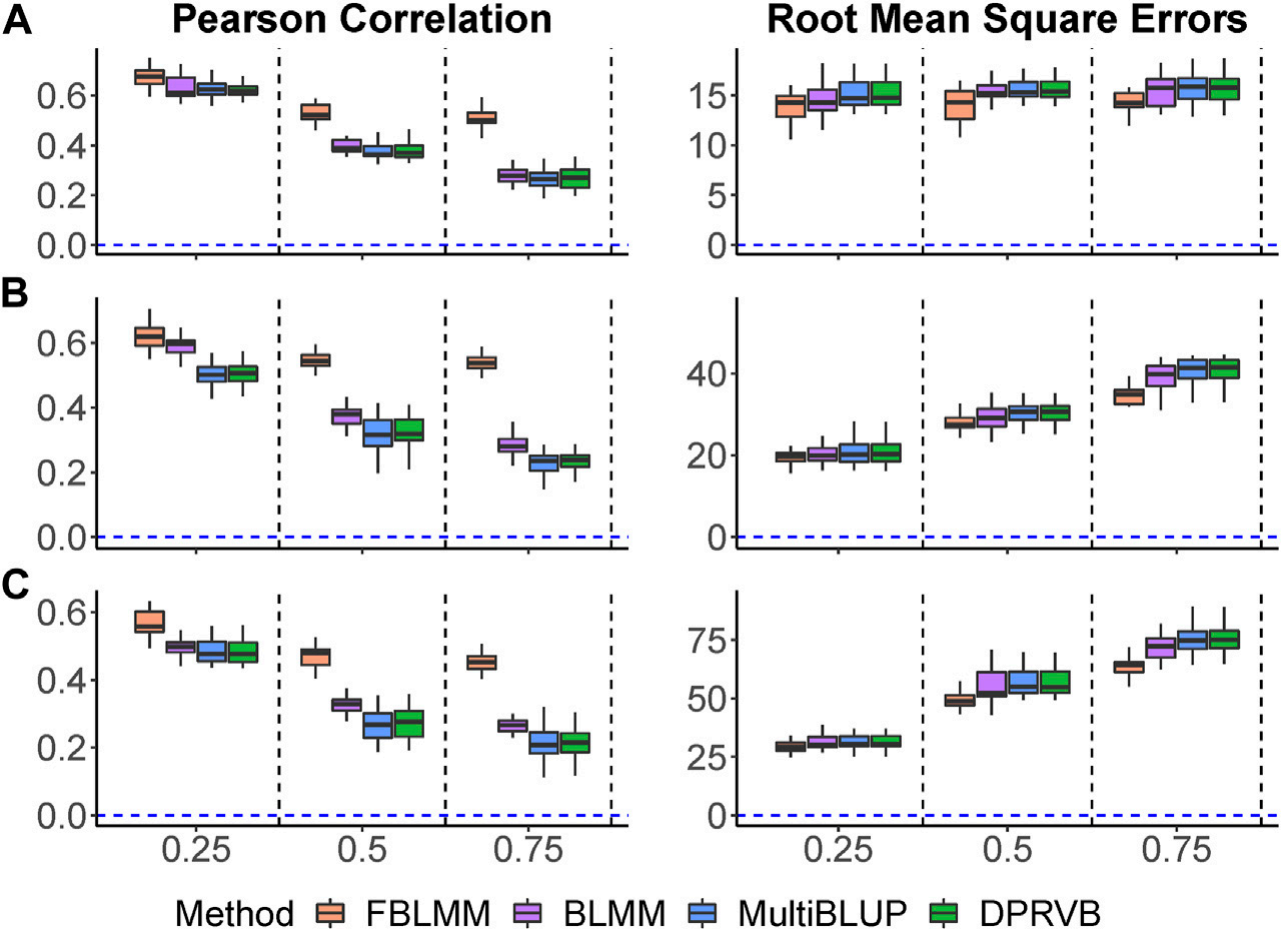
The comparison of prediction accuracy when outcomes are affected by unmeasured genetic variants. The total heritability is 60%, and the percentage of heritability accounted by unmeasured variants increases from 25% to 75%. **(A)**: Common variants affect the outcomes ( $w_{j}=1$ ). **(B)**: Rare variants affect the outcomes ( $w_{j}=dbeta{\left(MAF_{j},1,25\right)}^{2}$ ). **(C)**: Rare variants affect the outcomes $\left(w_{j}=\frac{1}{MAF_{j}\left(1-MAF_{j}\right)}\right)$ .

Pearson correlations and RMSEs are shown in Figure 3. As the proportion of genetic variance explained by unmeasured effects increases, the prediction accuracy for all methods decreases with FLBMM decreased the least. For FBLMM, it has robust performance across all settings. When outcomes are mainly caused by common genetic variants (Figure 3. A), FBLMM outperforms the other methods across all simulation settings and captures most of the heritability. This is mainly because FBLMM has an advantage in capturing the genetic effects from unmeasured variants via using the theoretical kinship coefficients. Not surprisingly, the performance of BLMM, MultiBLUP and DPRVB are very similar. When the disease outcomes were simulated under the assumption that rare variants had large contributions (Figure 3. B; Figure 3. C), FBLMM performs much better than the existing methods, and BLMM outperforms MultiBLUP and DPRVB. This is mainly because the weights in both FBLMM and BLMM are designed to capture the effects from rare variants. Therefore, FBLMM is expected to have a more robust performance through modeling familial correlations and up-weighting rare genetic variants.

### Outcome is affected by shared environmental and genetic factors

In this set of simulations, we evaluated the performance of FBLMM when outcomes were affected by both shared environmental and genetic factors. Three genes were randomly selected as causal, and outcomes were simulated under the following additive model:

$$Y=\alpha +\sum_{m=1}^{3}g_{m}+\epsilon ,$$ (9)

where $\alpha ∼N\left(0,K\sigma_{a}^{2}\right)$ and K is a block diagonal matrix. $g_{m}∼N\left(0,K_{m}\sigma_{m}^{2}\right)$ and $K_{m}=\left(G_{m}G_{m}^{T}\right)/p_{m}$ . Similar to previous section, among the three causal genes, we randomly set one of them as unmeasured in the data. We gradually increased the percentages of variabilities explained by the shared environmental and genetic effects from 20% to 60%, and both factors contributed equally (i.e., $\sigma_{a}^{2}=\sum_{m=1}^{2}\sigma_{m}^{3}$ ).

**FIGURE 4:**
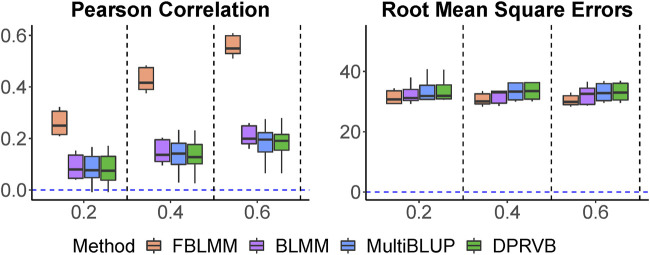
The comparison of prediction accuracy when outcomes are affected by both shared environmental factors and genetic variants, including both measured and unmeasured. The totally heritability increases from 20% to 60%, with both genetic and environmental factors contributing equally.

Pearson correlations and RMSEs are shown in Figure 4. As the proportion of variability explained by shared environmental and genetic factors increases, the proposed method tends to perform much better than the others. This is because FBLMM is designed to capture predictive effects from both genetic and environmental risk factors simultaneously, whereas the other methods have little ability to model them if they are not measured. Although it is well accepted that family history itself is an important predictor for many complex diseases, little efforts have been made to utilize information embedded in the family design. Our simulation shows that by using the design information, FBLMM can achieve robust performance and substantially improve the prediction models across a range of settings.

## Scenario 2: The impact of pedigree structures

In this set of simulations, we assessed the effects of pedigree structures on the performance of FBLMM. We considered the twin design (Supplementary Figure S1D), the trio design (Supplementary Figure S1E), and three-generation pedigree with mixed structures that include 24 avuncular, 30 double cousins, 42 grandparents and 278 sibling (Supplementary Figures S1F–I). We used Eq. 9 to simulate outcomes, where genetic variants on one causal gene is set as unmeasured. Let $g_{u}∼N\left(0,K_{u}\sigma_{u}^{2}\right)$ denote the cumulative predictive effects for the unmeasured gene, and Eq. 9 can be written as $Y=\alpha +\sum_{m=1}^{2}g_{m}+g_{u}+\epsilon$ .

We considered three types of disease models (Supplementary Table S1: both measured and unmeasured genetic variants have equally contributed to disease risk (i.e., $\sigma_{u}^{2}=\sum_{m=1}^{2}\sigma_{m}^{2}$ ); $S_{2}$ : shared environmental factors have major influences on disease risk, and measured genetic factors only make small contributions (i.e., $\sigma_{a}^{2}>\sum_{m=1}^{2}\sigma_{m}^{2}$ ); and $S_{3}$ : both genetic and shared environmental factors were considered with unmeasured genetic variants making major contributions (i.e., $\sigma_{u}^{2}>\sigma_{a}^{2}+\sum_{m=1}^{2}\sigma_{m}^{2}$ ). We set the total heritability for all disease models ranging from 20% to 60%, and the details of parameter settings for each disease model are summarized in Supplementary Table S1.

**FIGURE 5:**
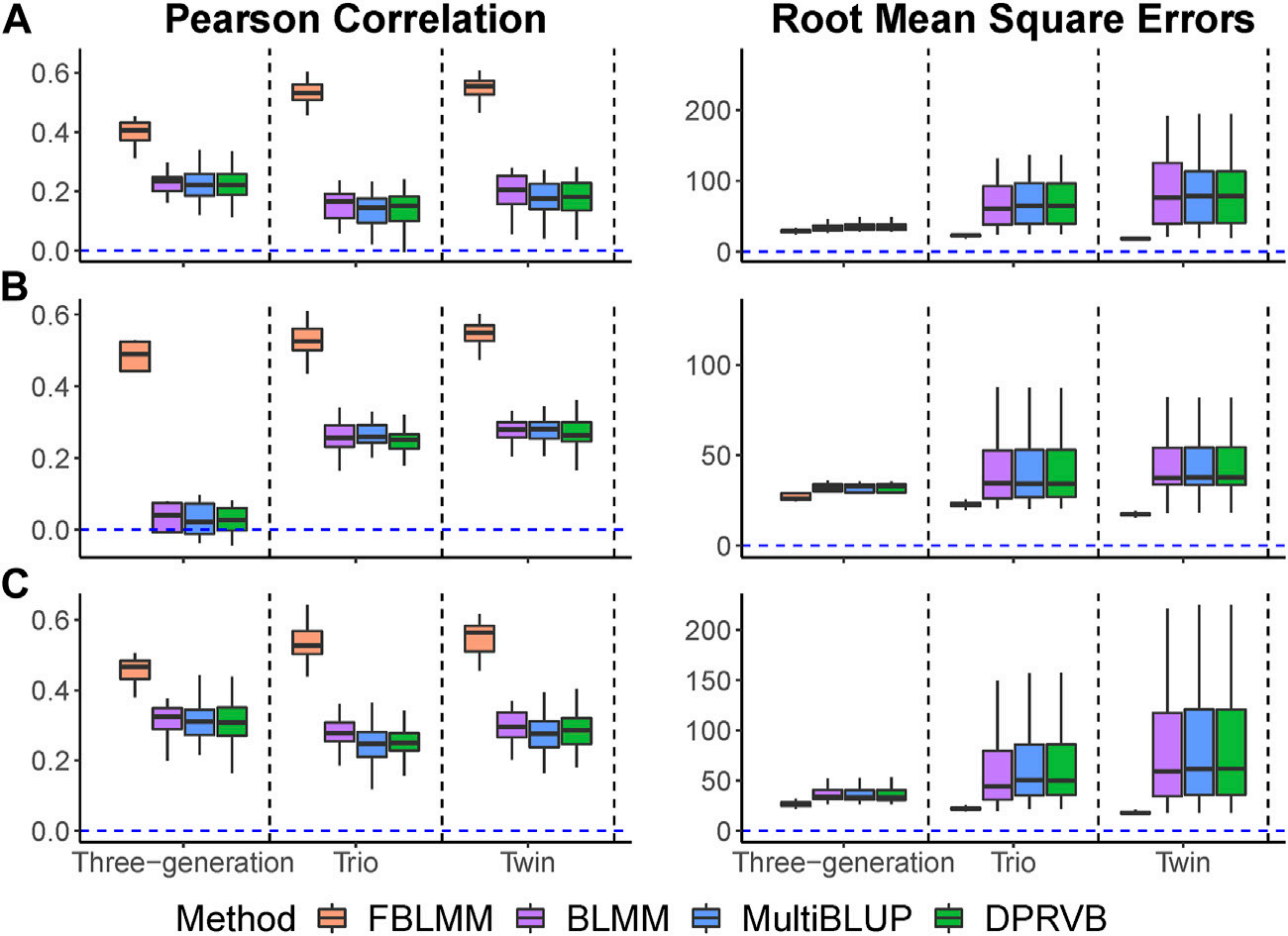
The comparison of prediction accuracy under different pedigree structures ( h=40% ). Three disease models are considered: **(A)** both measured and unmeasured genetic variants contributed to disease risk; **(B)** shared environmental and measured genetic factors affected outcomes; **(C)** all genetic variants (measured and unmeasured) and shared environmental factors contributed to disease risk.

The results when heritability is 40% are summarized in Figure 5, and the others (i.e., h=20% and h=60% ) are shown in Supplementary Figures S2, S3. Under disease model $S_{1}$ , where measured and unmeasured genetic variants contributed equally to disease risk, Figure 5A showed that the two-generation pedigree design has a higher prediction accuracy as compared to three-generation designs. This is mainly because relatives in two-generation pedigree have higher level of genetic relatedness than those that are far apart. Compared to existing methods, FBLMM worked the best across all pedigree structures under disease model $S_{1}$ and captured most of the heritability. Under disease model $S_{2}$ , where shared environmental factors mainly contributed to disease risk, Figure 5B showed that the existing methods (i.e., BLMM, MultiBLUP and DPR) have lower prediction performance as compared to FBLMM. FBLMM tended to perform similarly across all three pedigree structures considered, as shared environmental factors affect all individuals within the family in a similar fashion. Under disease model $S_{3}$ , where both unmeasured and environmental factors contribute significantly to the trait, two-generation pedigree structure tended to have higher prediction accuracy than the three-generation pedigree design (Figure 5C). Regardless of the pedigree structures and disease models considered, our proposed FBLMM always outperformed the other methods (i.e., BLMM, MultiBLUP and DPR). This indicates that FBLMM has robust performance in capturing the predictive effects from shared environmental and unmeasured genetic factors regardless of the pedigree structures. When the heritability is set to be 20% and 60%, the trend remains the same (Supplementary Figures S2, S3). By using the family design information, FBLMM has substantially enhanced the prediction accuracy, and the improvement is robust against various pedigree structures.

## Real data application

The proposed method is applied to predict aggressive behavior utilizing the dataset obtained from the Behavioral and Emotional Development in Children (TBED-C) study. TBED-C is a family-based twin study, aimed at discovering genetic factors that contribute to conduct problems in children (Burt and Klump, 2012). TBED-C recruited 1000 twins aged between 6 and 10 years from 500 twin families in Michigan, including 50% monozygous twins. DNA samples were collected from each pair of twins. The sequencing was performed using the Illumina Human Core Exome chip, which includes common variants, rare variants, mitochondrial DNA, and indels. Samples with missing rate >3% were excluded. Single nucleotide variants (SNVs) were removed if any of the following exclusion criteria was met: 1) call rate <98% and 2) a *p*-value for Hardy–Weinberg equilibrium test $<10^{-5}$ . After the quality control filtering, there are 957 samples and 513,886 SNVs remained for the analysis. Parents completed the child behavior checklist for each twin separately by rating a series of questionnaires, where children’s competencies, behavioral and emotional problems were assessed (Burt and Klump, 2012). Teacher(s) of each twin also completed the report form. Using the recommended approach (Burt and Klump, 2012), we assessed children’s aggressive behavior by averaging the raw scale scores from both the parents’ and teachers’ reports. The distribution of the aggressive scales (AGG, Mean=3.70 ; sd=3.59 ) is shown in Supplementary Figure S4.

First, to avoid over-fitting and the chance finding problems, 20% samples were randomly select for testing and the rest 80% was used for training. In the training dataset, we assessed the marginal significance for each marker using a linear hybrid model in the GCTA software package (Yang et al., 2011). Common variants with *p* values >0.1 were filtered out from risk prediction analysis. As a result, approximately 25,168 SNVs remained. This pre-selection aimed to prune a large number of predictors down drastically to a more manageable size, and improve computational speed. We applied all evaluated methods (i.e., FBLMM, BLMM, MulitBLUP and DPRVB) to the remaining genetic variants. Finally, we validated the trained FBLMM model using the test set. The prediction performance was evaluated using Pearson correlation and RMSE. This process was repeated 100 times.

**FIGURE 6:**
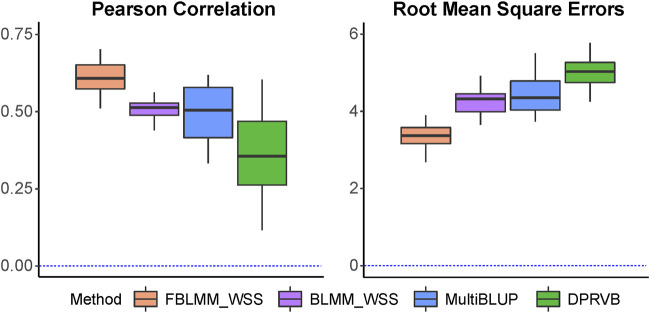
The comparison of prediction accuracy for aggressive scale.

Pearson correlations and the RMSEs are shown in Figure 6. Similar to results from simulations, Figure 6 shows FBLMM performed much better than the others. This clearly indicates that simply adjusting for relatedness among family members can overlook key information, leading to a less accurate risk prediction model. On contrary, utilizing information embedded in the family design can substantially improve prediction accuracy, as this makes the model more flexible to capture the predictive effects from unknown genetic and shared environmental risk factors.

## Discussion and conclusions

In this paper, we have developed a novel FBLMM method for risk prediction analysis on sequencing data obtained from family-based genetic studies. Fundamentally different from existing methods that adjust for family correlations, FBLMM utilizes this relatedness to further improve prediction accuracy. Specifically, it forms two surrogates, including a theoretical kinship coefficient matrix (i.e., $K^{g_{f}}$ ) and a block diagonal matrix (i.e., $K^{e_{f}}$ ), to capture effects from unmeasured genetic and shared environmental factors. In addition, FBLMM extends the BLMM method proposed by (Hai and Wen, 2020), and thus it inherits all the advantages in the BLMM method. For example, it infers its parameters using variational Bayes algorithm rather than the traditional MCMC, making it much more computationally efficient. Supplementary Table S2 provided the details of computational resources needed as the sample size and the number of variants increase. Furthermore, it can capture predictive effects from both common and rare variants, and easily accommodate various model assumptions (e.g., isolated large effects and infinitesimal model). It is worth noting that although we mainly focused on genetic variants, our proposed framework has the intrinsic capacity in modeling the predictive effects from important demographic variables, where their predictive effects can be selected and modelled through the fixed effects (i.e., Xβ ) in our model. For example, in addition to genetic information, we can add family history, age and gender into the fixed effect part (i.e., X ) of our model, and their predictive effects can be directly estimated by our proposed framework. Through simulation studies, we have shown that FBLMM can yield higher prediction accuracy than existing methods, and our analysis on Michigan Twin data has also showed that FBLMM can better predict AGG.

The importance of genetic and environmental factors in risk prediction has long been appreciated (Nilsson et al., 2004). Many previous studies have shown that a substantial amount of heritability can be explained by family information due to a combination of genetic factors and shared environmental conditions (Bermejo and Hemminki, 2005; Gim et al., 2017). The family information can be helpful in identifying sub-populations that are at high risk (MacInnis et al., 2011; So et al., 2011; Gim et al., 2017). Despite its clinical importance, few methods fully use this information when building risk prediction models based on high-dimensional genomic data obtained from family-based studies. Existing analytical methods are usually an extension of the models designed for population-based studies, and thus they tend to make the observations un-correlated before estimating the predictive effects from genetic variants (Meigs et al., 2008). While this most common practice can allow researchers to build a statistically valid risk prediction model using genomic data from family-based study designs, it overlooks important information embedded in the design, leading to a model with decreased prediction accuracy. In this study, one of the key features of our proposed model is that it utilizes the family design to improve prediction model, rather than simply adjusting for the correlations among family members. Based on the design information, we formed two surrogate measures, including a theoretical kinship coefficient matrix (i.e., $K^{g_{f}}$ ) and a block diagonal matrix (i.e., $K^{e_{f}}$ ), to capture the impacts of genetic and environmental risk factors. As shown in our simulation studies (Figure 2 to Figure 5) and the analysis of TBED-C dataset (Figure 6), we have shown that FBLMM have outperformed commonly used methods via using the design information, indicating our proposed method has the capacity to substantially improve prediction models for family-based studies.

Rare variants of large effects can play an important role in complex human diseases (Gaukrodger et al., 2005). It has been reported that the largest contributions to genetic risk of human diseases can come from rare variants (Mancuso et al., 2016; Hernandez et al., 2019). However, few family-based genetic studies are powerful enough to model these effects, primarily due to the lack of efficient analytical methods (McIntosh et al., 2016). We have recently developed BLMM for risk prediction studies using genomic data from population-based study designs (Hai and Wen, 2020), and BLMM has achieved an improved prediction accuracy through simultaneously considering both common and rare variants. Instead of modeling individual predictive effects that are hard to estimate for rare variants, BLMM models the cumulative predictive effects from a group of variables that include both common and rare variants. BLMM uses a WSS weight function that has been used in association analysis of sequencing data to address the contributions of rare variants (Wu et al., 2011), and this leads to an improvement for prediction studies. Our proposed FBLMM is built within the BLMM framework, and thus it inherits BLMM’s capacity in modeling rare variants. Same as BLMM, FBLMM uses the WSS function to up-weight the rare variants so that their predictive effects can be effectively captured. As shown in simulations, FBLMM can achieve better assessment, when outcomes were simulated under the assumption that rare variants significantly contribute to the risk (Figures 2B, C).

One of the limitations of our method is that it overlooks the contributions of non-additive effects, especially interactions. As indicated in existing literature (Weissbrod, Geiger, and Rosset, 2016), non-linear predictive effects (e.g., epistasis) widely exist. Therefore, it is important to incorporate non-additive effects into risk prediction models. A potential solution within the FBLMM framework is to kernelize the variance-covariance matrix of the random effect terms, so that the assumed relationships between predictors and outcomes can be non-linear. For example, similar to MKLMM (Weissbrod, Geiger, and Rosset, 2016), polynomial kernel of two degrees of freedom and the saturating pathway kernel can be used to capture non-linear predictive effects. This will be a future direction of our research.

In summary, we have proposed a Bayesian linear mixed model for risk prediction analysis on genomic data obtained from family-based study designs. Our proposed FBLMM extends the BLMM method, and thus it can not only capture the predictive effects from both common and rare variants, but also accommodate various disease model assumptions. In addition, using study design information, FBLMM forms two surrogates to model the predictive effects from unmeasured/unknown genetic and environmental risk factors, which substantially facilitates family-based prediction studies. The algorithm implementing our proposed method is available at https://github.com/yhai943/FBLMM.

## Data Availability

The R package is available at https://github.com/yhai943/FBLMM. The data presented in the study are deposited at figshare repository (https://figshare.com/s/02c32e50c5b7529c0fb5). The use of the original genotype data is subject to the approval from the TBED-C study team (https://msutwinstudies.com/msutr-data).
