# Supplementary material for: Bayesian linear mixed model with multiple random effects for family-based genetic studies
Source: Front Genet. 2023 Oct 19;14:1267704. doi: 10.3389/fgene.2023.1267704 (PMC10620972; doi:10.3389/fgene.2023.1267704)
Supplement: Supplementary file 1 [file Presentation1.PDF]

Supplementary Data of Bayesian linear mixed model with multiple random effects for family-based genetic studies

Yang Hai, Wenxuan Zhao, Qingyu Meng, Long Liu and Yalu Wen

A Variational Bayes for Parameter Inference

Recall $\boldsymbol{\xi} = (\boldsymbol{\beta}, \boldsymbol{\gamma}, \boldsymbol{U}^g, \boldsymbol{U}_{gf}, \boldsymbol{U}_{ef}, \boldsymbol{r}, \boldsymbol{\sigma}^2, \sigma^2_{gf}, \sigma^2_{ef}, \sigma^2_{\boldsymbol{\epsilon}})$ is all the parameter of interest, and the approximate posterior distribution of $\boldsymbol{\xi}$ is defined as

$$\begin{aligned} q(\boldsymbol{\xi}) =& q_{\boldsymbol{\beta}} \times \prod_{j=1}^{p} q_{\gamma_j} \times \prod_{m=1}^{M} q_{\boldsymbol{U}_m} \times \prod_{m=1}^{M} q_{r_m} \times q_{\boldsymbol{U}_{gf}} \times q_{\boldsymbol{U}_{ef}} \times \\ & \prod_{m=1}^{M} q_{\sigma^2_m} \times q_{\sigma^2_{gf}} \times q_{\sigma^2_{ef}} \times q_{\sigma^2_{\epsilon}}, \end{aligned}$$

where $q_{\boldsymbol{\beta}} = \mathcal{N}(\boldsymbol{M}_{\boldsymbol{\beta}}, \boldsymbol{S}_{\boldsymbol{\beta}})$; $q_{\gamma_j} = Bernoulli(\psi_j)$; $q_{\boldsymbol{U}_m} = \mathcal{N}(\boldsymbol{M}_m, \boldsymbol{S}_m)$; $q_{r_m} = Bernoulli(\phi_m)$; $q_{\boldsymbol{U}_{gf}} = \mathcal{N}(\boldsymbol{M}_{gf}, \boldsymbol{S}_{gf})$; $q_{\boldsymbol{U}_{ef}} = \mathcal{N}(\boldsymbol{M}_{ef}, \boldsymbol{S}_{ef})$; $q_{\sigma^2_m} = IG(a_m, b_m)$; $q_{\sigma^2_{gf}} = IG(a_{gf}, b_{gf})$; $q_{\sigma^2_{ef}} = IG(a_{ef}, b_{ef})$; and $q_{\sigma^2_{\epsilon}} = IG(a_{\epsilon}, b_{\epsilon})$. Each parameter of $\boldsymbol{\xi}$ can be estimated by using the coordinate ascent algorithm, and the following estimating equations are used to update the parameters.

Update $\boldsymbol{\beta}$: Recall the variation distribution for $q(\boldsymbol{\beta})$ is $q(\boldsymbol{\beta}) = \mathcal{N}(\boldsymbol{M}_{\boldsymbol{\beta}}, \boldsymbol{S}_{\boldsymbol{\beta}})$. The parameters $\boldsymbol{M}_{\boldsymbol{\beta}}$ and $\boldsymbol{S}_{\boldsymbol{\beta}}$ are updated according to the following estimating equations:

$$\begin{aligned} \boldsymbol{M}_{\boldsymbol{\beta}} =& E(\frac{1}{\sigma^2_{\boldsymbol{\epsilon}}}) \boldsymbol{S}_{\beta} E(\boldsymbol{\Gamma}) \boldsymbol{X}^T E(\boldsymbol{A}) \\ \boldsymbol{S}_{\boldsymbol{\beta}} =& \{E(\frac{1}{\sigma^2_{\boldsymbol{\epsilon}}})((\boldsymbol{X}^T\boldsymbol{X}) \circ \boldsymbol{\Omega}) + E(\frac{1}{\sigma^2_{\boldsymbol{\beta}}}\boldsymbol{I})\}^{-1}, \end{aligned} \tag{S1}$$

where $\boldsymbol{A} = \boldsymbol{y} - \sum_{m=1}^{M}(\boldsymbol{Z}_m(r_m\boldsymbol{I}_m\boldsymbol{U}_m)) - \boldsymbol{Z}_{ef}\boldsymbol{U}_{ef}$ - $\boldsymbol{Z}_{gf}\boldsymbol{U}_{gf}$; $\boldsymbol{\Omega} = \boldsymbol{\psi}\boldsymbol{\psi}^T + \boldsymbol{\Psi} \circ (\boldsymbol{I} - \boldsymbol{\Psi})$; $\boldsymbol{\Psi} = diag(\boldsymbol{\psi})$, $\boldsymbol{\psi} = E_q(\boldsymbol{\gamma})$; and $\circ$ denotes the Hadamard product.

Update γ_j**:** The variation distribution for $q(\gamma_j)$ is $q(\gamma_j) = Bernoulli(\psi_j)$, where $\psi_j = E(\gamma_j)$. To update γ_j, we use the following estimating equation:

$$\begin{aligned} logit(\psi_j) =& logit(\theta_0) - \frac{1}{2}E(\frac{1}{\sigma_\epsilon^2})\boldsymbol{X}_j^T\boldsymbol{X}_j(\boldsymbol{M}_{\boldsymbol{\beta}(j)}^T\boldsymbol{M}_{\boldsymbol{\beta}(j)} + \boldsymbol{S}_{\boldsymbol{\beta}_{(j,j)}}) \\ & -E(\frac{1}{\sigma_{\boldsymbol{\epsilon}}^2})\boldsymbol{X}_j^T\left(\boldsymbol{X}_{(-j)}\boldsymbol{\Gamma}_{(-j)}(\boldsymbol{M}_{\boldsymbol{\beta}(-j)}\boldsymbol{M}_{\boldsymbol{\beta}(j)} + \boldsymbol{S}_{\boldsymbol{\beta}_{(-j,j)}})\right) \\ & +E(\frac{1}{\sigma_{\boldsymbol{\epsilon}}^2})\boldsymbol{X}_j^T(E(\boldsymbol{A})\boldsymbol{M}_{\boldsymbol{\beta}(j)}), \end{aligned} \tag{S2}$$

where $logit(\psi_j) = \log(\frac{\psi_j}{1-\psi_j})$; $\boldsymbol{M}_{\boldsymbol{\beta}(j)}$ is the jth component of $\boldsymbol{M}_{\boldsymbol{\beta}}$; $\boldsymbol{M}_{\boldsymbol{\beta}(-j)}$ is the whole vector of $\boldsymbol{M}_{\boldsymbol{\beta}}$ except the single component at index j; $\boldsymbol{X}_{(-j)}$ and $\boldsymbol{\Gamma}_{(-j)}$ are the matrices of $\boldsymbol{X}$ and $\boldsymbol{\Gamma}$ except the jth column; and $\boldsymbol{S}_{\boldsymbol{\beta}_{(-j,j)}}$ is jth column of $\boldsymbol{S}_{\boldsymbol{\beta}}$ without the jth component.

Update $\boldsymbol{U}_m$**:** The variational distribution for $q(\boldsymbol{U}_m)$ is $q(\boldsymbol{U}_m) = \mathcal{N}(\boldsymbol{M}_m, \boldsymbol{S}_m)$, with mean $\boldsymbol{M}_m$ and variance $\boldsymbol{S}_m$. Therefore, the parameters are updated as:

$$\begin{aligned} \boldsymbol{M}_m =& E(\frac{1}{\sigma_{\boldsymbol{\epsilon}}^2})\boldsymbol{S}_m(E(\gamma_m)\boldsymbol{I}_m)\boldsymbol{Z}_m^T E(\boldsymbol{B}_m) \\ \boldsymbol{S}_m =& \{E(\frac{1}{\sigma_{\boldsymbol{\epsilon}}^2})((\boldsymbol{Z}_m^T\boldsymbol{Z}_m) \circ (E(r_m)\boldsymbol{I}_m)) + E(\frac{1}{\sigma_m^2}\boldsymbol{I}_m)\}^{-1} \end{aligned}, \tag{S3}$$

where $\boldsymbol{B}_m = \boldsymbol{y} - \boldsymbol{X}\boldsymbol{\Gamma}\boldsymbol{\beta} - \sum_{i \neq m}(\boldsymbol{Z}_i(r_i\boldsymbol{I}_i\boldsymbol{U}_i)) - \boldsymbol{Z}_{ef}\boldsymbol{U}_{ef}$ - $\boldsymbol{Z}_{gf}\boldsymbol{U}_{gf}$.

Update r_m**:** The variation distribution for $q(r_m)$ is $q(r_m) = Bernoulli(\phi_m)$, where $\phi_m = E(r_m)$. Hence, the estimating equation for updating r_m is

$$\begin{aligned} logit(\phi_m) =& logit(\theta_1) - \frac{1}{2\sigma_{\boldsymbol{\epsilon}}^2}(\boldsymbol{M}_m^T\boldsymbol{Z}_m^T\boldsymbol{Z}_m\boldsymbol{M}_{\boldsymbol{u}_m} \\ & +tr(\boldsymbol{Z}_m^T\boldsymbol{Z}_m\boldsymbol{S}_m)) + \frac{1}{2\sigma_{\boldsymbol{\epsilon}}^2}E(\boldsymbol{B}_m^T)\boldsymbol{Z}_m\boldsymbol{M}_m\} \end{aligned}. \tag{S4}$$

Update σ_m^2: The variational distribution for $q(\sigma_m^2)$ is $q(\sigma_m^2) = IG(a_m, b_m)$. Therefore, the parameters are updated by

$$\begin{aligned} a_m =& \frac{n}{2} + a_1 \\ b_m =& E(\frac{1}{2}\boldsymbol{U}_m^T \boldsymbol{U}_m) + b_1 \end{aligned}. \tag{S5}$$

Update $\boldsymbol{U}_{gf}$: The variational distribution for $q(\boldsymbol{U}_{gf})$ is $q(\boldsymbol{U}_{gf}) = \mathcal{N}(\boldsymbol{M}_{gf}, \boldsymbol{S}_{gf})$, with mean $\boldsymbol{M}_{gf}$ and variance $\boldsymbol{S}_{gf}$. Therefore, the parameters are updated as:

$$\begin{aligned} \boldsymbol{M}_{gf} =& E(\frac{1}{\sigma_{\boldsymbol{\epsilon}}^2})\boldsymbol{S}_{gf}(E(r_{gf})\boldsymbol{I})\boldsymbol{Z}_{gf}^T E(\boldsymbol{B}_{gf}) \\ \boldsymbol{S}_{gf} =& \{E(\frac{1}{\sigma_{\boldsymbol{\epsilon}}^2})((\boldsymbol{Z}_{gf}^T \boldsymbol{Z}_{gf})) + E(\frac{1}{\sigma_{gf}^2} I)\}^{-1} \end{aligned}, \tag{S6}$$

where $\boldsymbol{B}_{gf} = \boldsymbol{y} - \boldsymbol{X}\boldsymbol{\Gamma}\boldsymbol{\beta} - \sum_{m=1}^{M}(\boldsymbol{Z}_m(r_m \boldsymbol{I}_m \boldsymbol{U}_m)) - \boldsymbol{Z}_{ef}\boldsymbol{U}_{ef}$.

Update σ_{gf}^2: The variational distribution for $q(\sigma_{gf}^2)$ is $q(\sigma_{gf}^2) = IG(a_{gf}, b_{gf})$, and thus the parameters are updated by

$$\begin{aligned} a_{gf} =& \frac{n}{2} + a_1 \\ b_{gf} =& E(\frac{1}{2}\boldsymbol{U}_{gf}^T \boldsymbol{U}_{gf}) + b_1 \end{aligned}. \tag{S7}$$

Update $\boldsymbol{U}_{ef}$: The variational distribution for $q(\boldsymbol{U}_{ef})$ is $q(\boldsymbol{U}_{ef}) = \mathcal{N}(\boldsymbol{M}_{ef}, \boldsymbol{S}_{ef})$, with mean $\boldsymbol{M}_{ef}$ and variance $\boldsymbol{S}_{ef}$. Therefore, the parameters are updated as:

$$\begin{aligned} \boldsymbol{M}_{ef} =& E(\frac{1}{\sigma_{\boldsymbol{\epsilon}}^2})\boldsymbol{S}_{ef}(E(r_{ef})\boldsymbol{I})\boldsymbol{Z}_{ef}^T E(\boldsymbol{B}_{gf}) \\ \boldsymbol{S}_{ef} =& \{E(\frac{1}{\sigma_{\boldsymbol{\epsilon}}^2})((\boldsymbol{Z}_{ef}^T \boldsymbol{Z}_{ef}) + E(\frac{1}{\sigma_{ef}^2} I)\}^{-1}, \end{aligned} \tag{S8}$$

where $\boldsymbol{B}_{ef} = \boldsymbol{y} - \boldsymbol{X}\boldsymbol{\Gamma}\boldsymbol{\beta} - \sum_{m=1}^{M}(\boldsymbol{Z}_m(r_m \boldsymbol{I}_m \boldsymbol{U}_m)) - \boldsymbol{Z}_{gf}\boldsymbol{U}_{gf}$.

Update σ_{ef}^2: The variational distribution for $q(\sigma_{ef}^2)$ is $q(\sigma_{ef}^2) = IG(a_{ef}, b_{ef})$. Therefore, we have

$$\begin{aligned} a_{ef} =& \frac{n}{2} + a_1 \\ b_{ef} =& E(\frac{1}{2}\boldsymbol{U}_{ef}^T\boldsymbol{U}_{ef}) + b_1 \end{aligned}. \tag{S9}$$

Update σ_{ϵ}^2: The variational distribution for $q(\sigma_{\epsilon}^2)$ is $q(\sigma_{\epsilon}^2) = IG(a_{\epsilon}, b_{\epsilon})$. The parameters a_{ϵ} and b_{ϵ} are updated according to following equations:

$$\begin{aligned} a_{\epsilon} =& \frac{n}{2} + a_0 \\ b_{\epsilon} =& E(\frac{1}{2}E(\boldsymbol{C}^T\boldsymbol{C})) + b_0 \end{aligned}, \tag{S10}$$

where $\boldsymbol{C} = \boldsymbol{y} - \boldsymbol{X}\boldsymbol{\Gamma}\boldsymbol{\beta} - \sum_{m=1}^{M}(\boldsymbol{Z}_m(r_m\boldsymbol{I}_m\boldsymbol{U}_m)) - \boldsymbol{Z}_{gf}\boldsymbol{U}_{gf}$ - $\boldsymbol{Z}_{ef}\boldsymbol{U}_{ef}$.

B Supplementary Tables

Table S1: Overview of parameter setting for simulation 2.

	$h = 20\%$			$h = 40\%$			$h = 60\%$		
	σ_a^2	σ_u^2	$\sum_{m=1}^{2} \sigma_m^2$	σ_a^2	σ_u^2	$\sum_{m=1}^{2} \sigma_m^2$	σ_a^2	σ_u^2	$\sum_{m=1}^{2} \sigma_m^2$
Model S_1	0	0.1	0.1	0	0.2	0.2	0	0.3	0.3
Model S_2	0.1	0	0.1	0.3	0	0.1	0.5	0	0.1
Model S_3	0.05	0.1	0.05	0.05	0.3	0.05	0.05	0.5	0.05

Table S2: The computational time (Intel Xeon Processor E5-2695 v4 2.1 GHz, dual-core) and memory as the number of samples and variants increases.

No. of Samples	No. of Variants	times(min)	memory(Gb)
500	1000	2	1
	5000	4	1.5
	10000	4	2
	50000	14	4
1000	1000	2	1
	5000	5	2.5
	10000	10	4
	50000	20	6
2000	1000	4	2
	5000	8	3
	10000	14	6
	50000	30	10

C Supplementary Figures

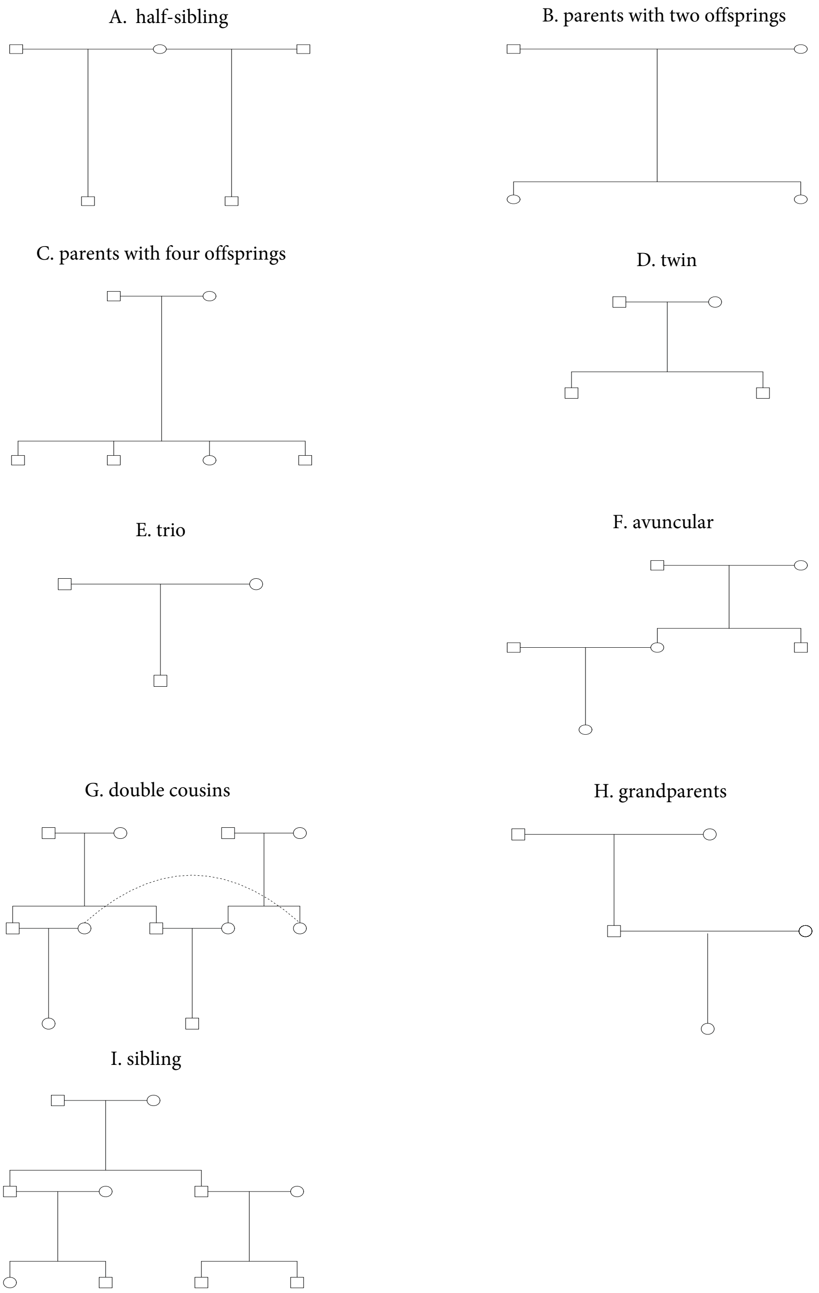

Figure S1: Pedigree structures used in this study.

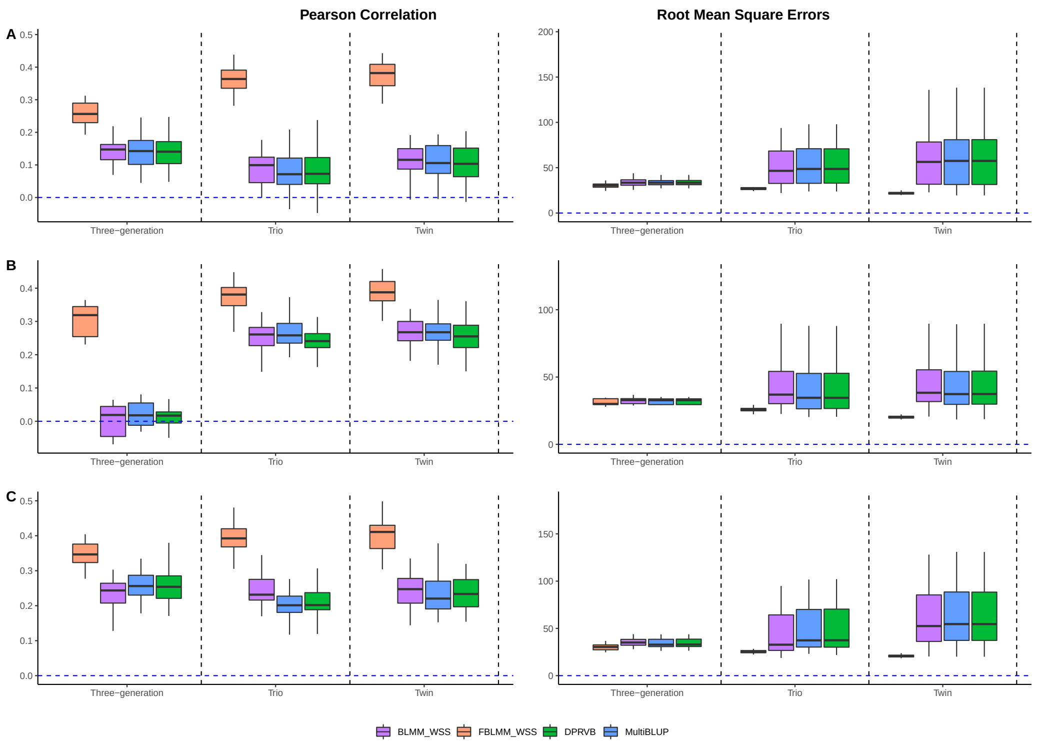

Figure S2: Comparison of prediction performance under different pedigree structures ($h = 20\%$). Three disease models have been considered: (A) both measured and unmeasured genetic variants contributed to disease risk; (B) shared environmental and measured genetic factors affected outcomes; (C) all genetic variants (measured and unmeasured) and shared environmental factors contributed to disease risk.

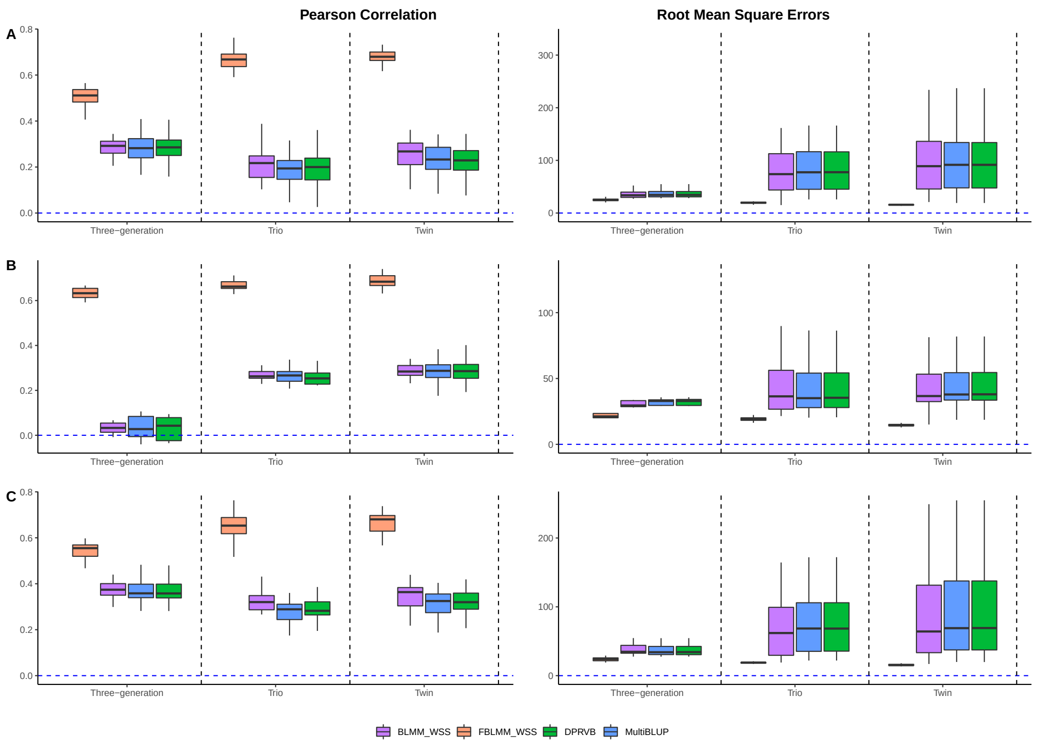

Figure S3: Comparison of prediction performance under different pedigree structures ($h = 60\%$). Three disease models have been considered: (A) both measured and unmeasured genetic variants contributed to disease risk; (B) shared environmental and measured genetic factors affected outcomes; (C) all genetic variants (measured and unmeasured) and shared environmental factors contributed to disease risk.

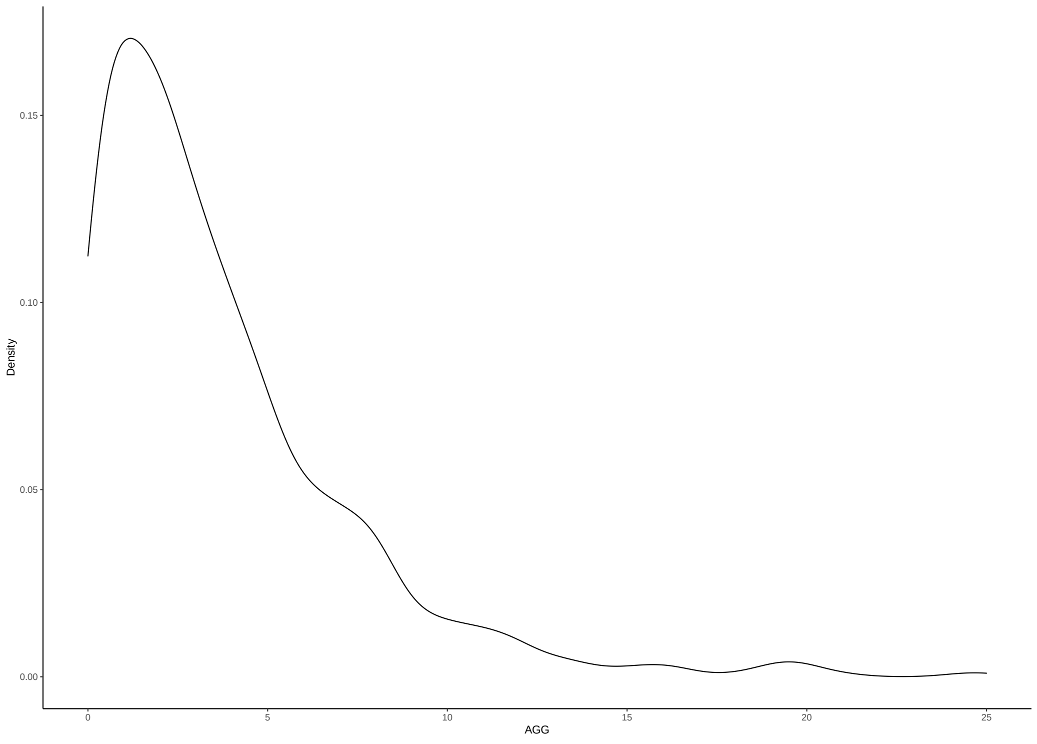

Figure S4: Distributions for AGG.
